# The CARDS toxin of *Mycoplasma pneumoniae* induces a positive feedback loop of type 1 immune response

**DOI:** 10.3389/fimmu.2022.1054788

**Published:** 2022-12-01

**Authors:** Ting Wang, Huiming Sun, Zhitao Lu, Wujun Jiang, Ge Dai, Li Huang, Meijuan Wang, Canhong Zhu, Yuqing Wang, Chuangli Hao, Yongdong Yan, Zhengrong Chen

**Affiliations:** ^1^Department of Respiratory Medicine, Children’s Hospital of Soochow University, Suzhou, China; ^2^Department of Pediatrics, Zhangjiagang No.1 People’s Hospital, Affiliated Hospital of Soochow University, Suzhou, China

**Keywords:** *Mycoplasma pneumoniae*, CARDS toxin, type 1 immune response, CXCL9, positive feedback

## Abstract

**Background:**

Within the past 3-5 years, *Mycoplasma pneumoniae* has become a major pathogen of community-acquired pneumonia in children. The pathogenic mechanisms involved in *M. pneumoniae* infection have not been fully elucidated.

**Methods:**

Previous protein microarray studies have shown a differential expression of CXCL9 after *M. pneumoniae* infection. Here, we conducted a hospital-based study to explore the clinical significance of the type 1 immune response inflammatory factors interferon (IFN)-γ and CXCL9 in patients with *M. pneumoniae* pneumonia (MPP). Then, through *in vitro* experiments, we explored whether CARDS toxin stimulated F-DCs (dendritic cells incubated with Flt3L) to promote Th-cell differentiation; we also investigated the IFN-γ-induced CXCL9 secretion pathway in macrophages and the role of CXCL9 in promoting Th1 cell migration.

**Results:**

The CXCL9 expression level was upregulated among patients with a higher fever peak, fever duration of greater than 7 days, an imaging manifestation of lobar or segmental, or combined pleural effusion (*P*<0.05). The peripheral blood levels of IFN-γ and CXCL9, which were higher in patients than in the healthy control group, were positively correlated with each other (*r*=0.502, *P*<0.05). In patients, the CXCL9 expression level was significantly higher in the bronchoalveolar lavage fluid (BALF) than in the peripheral blood, and the BALF CXCL9 expression level was higher than that in the healthy control group (all *P*<0.05). Our flow cytometry analysis revealed that M1-phenotype macrophages (CD16**^+^** CD64**^+^** CD163^−^) were predominant in the BALF from children with MPP. In *in vitro* experiments, F-DCs stimulated with CARDS toxin promoted the differentiation of CD4**^+^** IFN-γ**^+^** Th (Th1) cells (*P*<0.05). Moreover, IFN-γ induced high levels of CXCL9 expression in M1-type macrophages in a dose-dependent and time-dependent manner. Additionally, macrophages transfection with STAT1-siRNA-1 downregulated the expression of CXCL9 (*P*<0.05), and CXCL9 promoted Th1 cell migration (*P*<0.05).

**Conclusions:**

Our findings suggest that CARDS toxin induces a type 1 immune response positive feedback loop during *M. pneumoniae* infection; this putative mechanism may be useful in future investigations of immune intervention approaches for *M. pneumoniae* pneumonia.

## Introduction

Acute respiratory tract infections are the most common and frequently occurring childhood diseases worldwide, particularly in children aged <5 years (1). *Mycoplasma pneumoniae* is a major respiratory tract pathogen in children (2, 3). The clinical manifestations of *M. pneumoniae* infections are diverse, in terms of both respiratory disease and a wide range of extrapulmonary manifestations (4, 5). The incidence of community-acquired pneumonia (CAP) caused by *M. pneumoniae* is characterized by cyclical epidemics at intervals of 3 to 7 years (6); up to 40% of CAP cases can be attributed to *M. pneumoniae* (7). In recent years, the annual incidence of *M. pneumoniae* pneumonia (MPP) has been increasing and become more severe, with about 5% requiring intensive care unit treatment (8). Severe *M. pneumoniae* pneumonia (SMPP) is characterized by massive pleural effusion, acute respiratory distress syndrome (ARDS), pulmonary consolidation, pulmonary fibrosis, obstructive bronchitis, and even life-threatening pulmonary sequelae, such as bronchiectasis, atelectasis, occlusive bronchitis, etc. Some children present with necrotizing pneumonia (NP) (9). After treatment with macrolide antibiotics for ≥7 days, the clinical manifestations in some children with MPP (usually children with SMPP) are not relieved and the lung imaging findings gradually worsen; these patients are diagnosed with refractory *M. pneumoniae* pneumonia (RMPP) (10).

There are no specific clinical symptoms during the early stage of *M. pneumoniae* infection in children; in the absence of clear clinical signs, affected patients can develop SMPP or RMPP (11). Systemic inflammatory responses and immune disorders have important roles in the occurrence, development, and prognosis of SMPP or RMPP; the influence of macrolide antibiotic resistance is increasing (12, 13). Therefore, considerable global research attention has been focused on the immunological pathogenesis of *M. pneumoniae* infection, as well as efforts for early identification and intervention.

The network of inflammatory interactions formed by innate and adaptive immunity has an important role in MPP and is closely related to disease severity (12). It’s mainly composed of types 1, 2, and 3 immune responses causing cytokine “waves”. The type 1 immune response mainly involves Th1 cells, interferon (IFN)-γ, and M1 macrophages (Mφs), which exert immune effector functions. The expression level of the Th1 cytokine IFN-γ is closely associated with the severity of MPP and the degree of recovery (14). M1-type Mφs can be activated by IFN-γ (15); numerous inflammatory factors (e.g., interleukin [IL]-1β, IL-6, tumor necrosis factor-α, and CXCL9) are then released. Chemokine CXCL9, also known as the IFN-γ-induced monokine, can recruit leukocytes to sites of inflammation. CARDS toxin is a virulence factor that stimulates excessive immuno-inflammatory responses after *M. pneumoniae* infection; its functions include adenosine diphosphate-ribosylation and vacuolization (16, 17).Inhibition of CARDS toxin is expected to reduce MPP severity, and CARDS toxin may be useful as a vaccine antigen (18). Therefore, the specific regulatory mechanism of IFN-γ/CXCL9 and CARDS toxin remains unclear, which has important research implications for MPP.

In the present study, we collected peripheral blood and bronchoalveolar lavage fluid (BALF) from children with MPP, conducting a hospital-based study to explore the clinical significance of the type 1 immune response inflammatory factors IFN-γ/CXCL9; through *in vitro* assays, we explored whether CARDS toxin promote Th-cell differentiation and the IFN-γ-induced CXCL9 secretion pathway in macrophages. Our findings may be useful in early identification, guidance regarding treatment and prognosis, and future investigations of targeted treatment and immune intervention approaches for MPP.

## Materials and Methods

### Patients 

This study included patients who were diagnosed with MPP from July 2019 to October 2021 at Children’s Hospital of Soochow University (N=140); all diagnoses were confirmed by both MP-immunoglobulin (IgM) positivity and the presence of MP-DNA (≥1.0×10^4^ copies/ml) in nasopharyngeal aspirates(NPA) or BALF, as measured by real-time quantitative polymerase chain reaction (qPCR) (19). The patients’ ages ranged from 17 months to 16 years; their clinical manifestations included fever, cough, tachypnea, chest retractions, abnormal auscultatory findings, and radiologic evidence of CAP.

Among MPP patients, 117 cases were selected as experimental group A to study the correlation of CXCL9 expression level in peripheral blood; while 23 cases were selected as experimental group B and measured IFN-γ and CXCL9 in peripheral blood and BALF; to further study the correlation between IFN-γ/CXCL9 and immune inflammatory damage in the lung.

Cases met one or more of the exclusion criteria were excluded (a): co-infection (b); clinical and imaging features indicative of fungal pneumonia (c); presence of congenital respiratory diseases, and abnormalities in other systems (e.g., heart, liver, kidney, and blood) (d); >3 days of treatment with glucocorticoids (20) and/or azithromycin before admission. To exclude co-infections, seven respiratory virus antigen tests (influenza A and B; parainfluenza 1, 2, and 3; respiratory syncytial virus; and adenovirus) and other pathogen tests (human bocavirus; human rhinovirus; human metapneumovirus; and chlamydia pneumoniae) were conducted; the results were negative. The results of bacterial cultures of NPA and BALF were also negative.

Peripheral blood and BALF were also collected from 18 children who underwent emergency surgery for foreign bodies in the ENT department of our hospital during the same period with 23 cases of MPP children; these children served as a control group. The inclusion criteria were as follows: no respiratory tract infection within the previous 4 weeks, no chronic lung disease or bronchopulmonary malformation, and no history of treatment with hormones or immunosuppressive agents.

The studies involving human participants were reviewed and approved by the Ethics Committee of the Children’s Hospital of Soochow University (2019LW014) on July 24, 2019. Written informed consent to participate in this study was provided by the participants’ legal guardian/next of kin. All participant data were anonymized prior to analysis.

### Collection of clinical data

All patients’ age and sex were recorded. In the experimental group A, the following clinical data were recorded: duration of fever, fever peak, and imaging manifestations. Peripheral blood samples obtained within 24 h of admission were used for measurements of CXCL9 and specific antibodies to *M. pneumoniae*.

In the experimental group B, the following clinical data were also recorded: durations of hospital stay and fever, fever peak, and laboratory test data on admission (**Table 1**). Peripheral blood samples obtained within 24 h of admission were also used for measurements of complete blood counts, C-reactive protein (CRP), lactate dehydrogenase (LDH), immunoglobulins (IgA/IgG/IgM), lymphocyte subsets, specific antibodies to *M. pneumoniae*, IFN-γ and CXCL9. Flexible fiber bronchoscopy and bronchoalveolar lavage were performed in accordance with existing guidelines (21). BALF was gently aspirated, collected, and prepared for detection of the protein concentrations of IFN-γ and CXCL9.

**Table 1:** Demographic data and clinical characteristics in experimental group B.

Clinical parameters	MPP cases	Controls		Blood-		BALF-	
	(experimental group B, n=23)	(n=18)	*p*	CXCL9		CXCL9	
				*r*	*P*	*r*	*P*
Age, years	4.90(3.80,7.00)	2.25(1.90,2.73)	0.00^*^	N/A	N/A	N/A	N/A
Male, n (%)	8(34.78%)	10(55.56%)	0.183	N/A	N/A	N/A	N/A
Hospital stay, days	9.13±2.26	N/A		-0.04	0.86	-0.33	0.13
Duration of fever, days	6.78±2.76	N/A		-0.43	0.040^*^	0.44	0.036^*^
Peak of fever (°C)	39.36±0.59	N/A		-0.51	0.51	0.16	0.48
WBC, 10^9^/L	7.59±3.41	N/A		0.3	0.17	0.19	0.4
N (%)	53.23±17.10	N/A		0.54	0.009^*^	0.17	0.43
PLT, 10^9^/L	334.30±137.68	N/A		0.15	0.49	0.11	0.62
EOS (%)	1.95±1.50	N/A		-0.34	0.11	0.42	0.048^*^
CRP, mg/L	12.71±12.66	N/A		-0.06	0.77	0.05	0.82
LDH, IU/L	384.58±125.10	N/A		0.33	0.13	0.06	0.77
IgA, g/L	1.02±0.53	N/A		-0.17	0.44	0.28	0.2
IgG, g/L	9.31±2.36	N/A		0.01	0.96	0.27	0.21
IgM, g/L	1.38±0.41	N/A		0.36	0.09	0.06	0.79
CD3**^+^**, %	71.79±5.85	N/A		-0.23	0.33	0.13	0.6
CD3**^+^** CD4**^+^**, %	41.79±5.96	N/A		0.09	0.71	-0.28	0.23
CD3**^+^** CD8**^+^**, %	27.88±5.79	N/A		-0.24	0.32	0.29	0.21
CD3**^-^** CD (16**^+^** 56**^+^**), %	8.68±4.04	N/A		-0.14	0.56	0.11	0.63
CD3**^-^** CD19**^+^**, %	18.45±5.67	N/A		0.39	0.09	-0.27	0.25

*P < 0.05.

### Real-time fluorescent qPCR for *M. pneumoniae* in NPA or BALF

Nucleic acid extraction and qPCR for the detection of *M. pneumoniae* 16S rDNA were performed as previously described (17, 21). Briefly, samples (NPA/BALF) were shaken for 30s, then centrifuged at 12,000×g for 5 min. Subsequently, the sediment was collected and DNA was extracted from 400-μL of each sample. The primers for *M. pneumoniae* 16S rDNA were as follows: forward: 5′-GCAAGGGTTCGTTATTTG-3′; reverse: 5′-CGCCTGCGCTTGCTTTAC-3′ (amplicon size: 380 bp).

Real-time qPCR was performed using the iQ5TM BIO-icycler (Bio-Rad, Hercules, CA, USA). The PCR conditions were as follows: 37°C for 2 min; initial denaturation at 94°C for 10 min, followed by 40 cycles of denaturation at 94°C for 10s, annealing at 55°C for 30s, and extension at 72°C for 40s. After amplification, a computer connected to the instrument automatically analyzed the results, then expressed the test results as Ct values. For samples with a Ct value <38, a quantitative reagent (Aikang Co., Ltd., Hangzhou, China) was used for further quantitative determination.

### Detection of IFN-γ and CXCL9 by enzyme-linked immunosorbent assays

IFN-γ and CXCL9 were measured in peripheral blood or/and BALF samples from MPP and control groups using the appropriate commercial enzyme-linked immunosorbent assay kits, in accordance with the manufacturer’s instructions. Enzyme-linked immunosorbent assay kits were purchased from Xuguang kexing Biotechnology Co., Ltd. (Suzhou, China).

### Flow cytometry analysis of cell counts and Mφ phenotypes in BALF from children with MPP

Flow cytometry was used to determine cell counts of Mφs, and lymphocytes in BALF from children with MPP, using flow cytometry equipment (Beckman Coulter, Miami, FL). The forward scatter (FSC) value represents cell volume; a larger FSC value indicated greater cell volume. The side scatter (SSC) value represents cell granularity; a larger SSC value indicated higher granularity. Cells with greater volume and higher granularity were considered neutrophils; cells with less volume and lower granularity were considered lymphocytes; monocytes (Mφs) were in between the two types of cells; then, Mφ phenotypes were determined by flow cytometry.

### Protein microarray

Protein microarrays enable determination of the protein-binding specificities of multiple analytes in solution; they are versatile tools for high-throughput analyses of the human proteome (22). In a preliminary experiment, six blood samples from the SMPP group, four blood samples from the MPP group, and five blood samples from the control group were analyzed by Ray Biotech Co., Ltd. (Guangzhou, China), which provided all reagents and instruments.

Multiple protein molecules were immobilized on a solid support in a predetermined arrangement to form a microarray. Samples were incubated with the protein chip; after specific binding reactions occurred, the components that were not bound to the protein on the chip were removed by washing. Fluorescently labeled antibodies were used for secondary incubation; the fluorescence value of each point on the chip was analyzed using a commercial fluorescence scanning analysis instrument and software.

### Animals and F-DCs (DCs incubated with Flt3L) induction

The animal study was reviewed and approved by the Ethics Committee of Soochow University (SUDA20200510A02) on May 10, 2020. C57BL/6 mice (age, 4–5 weeks; weight, 20±2g) were purchased from the Laboratory Animal Center of Soochow University (Suzhou, China). All animals were housed in separate cages under constant temperature (25±1°C) and humidity (50%) with 12-h light-dark cycles; they had free access to food and water.

Bone marrow mononuclear cells (BM-MNCs) were isolated from C57BL/6 mouse bone marrow *via* density gradient centrifugation. BM-MNCs were cultured in 24-well plates with Mouse Steady-State Dendritic Cell Culture Kit (Dongling Biotechnology Co., Ltd., Suzhou, China); the cell density was adjusted to 1.5×10^6^ cells/ml. Flt3L (Jinsirui Biotechnology Co., Ltd., Nanjing, China) was added to each well at a final concentration of 200 ng/ml; we obtained suspension cultured cells after 9 days of incubation (23), which were DCs with diverse phenotypic characteristics (i.e., F-DCs). Cultures were incubated at 37°C in a humidified atmosphere containing 5% CO_2_ in air.

### Construction of recombinant CARDS toxin

Recombinant CARDS toxin was constructed from Sino Biological Co., Ltd. (Beijing, China) and its biological function was verified as previously described (17, 21). According to the manufacturer’s instructions, the recombinant CARDS toxin was expressed in insect cells, and the insect cell expression system belonged to the eukaryotic cell expression system. From the NCBI database, we obtained the full-length gene sequence and protein sequence of MPN372, which coded CARDS Toxin. The optimized MPN372 gene sequence was cloned into the pFastBac donor plasmid vector for virus packaging. High-Five cells in logarithmic growth phase were infected with high titer recombinant virus to express the target protein, which was further purified by nickel column.

### Use of CARDS toxin to stimulate F-DCs for the regulation of Th cell differentiation *in vitro*

CD4**^+^** T cells (1×10^5^ cells/ml) were isolated from mouse spleens in accordance with the instructions of the Mouse Naïve CD4**^+^** T Cell Isolation Kit (STEMCELL Technologies, Vancouver, Canada). Labeled cells were isolated using EasySep magnets without a column, and the target cells were transferred to new tubes. Naïve CD4**^+^** T cells from the spleen were negatively selected using magnetic beads. Flow cytometry equipment was described as previously.

F-DCs were screened for phenotype by flow cytometry and the cell concentration was adjusted to 1×10^5^ cells/well. F-DCs incubated with cell growth medium were used as the negative control group; F-DCs incubated with CARDS toxin (10 ng/ml) were used as the experimental group. Both groups of cells were co-incubated with naïve CD4**^+^** T cells (1:4 ratio of DCs to T cells) for 24 h at 37°C with 5% CO_2_. The numbers of CD4**^+^** IFN-γ**^+^** Th, CD4**^+^** IL-4**^+^** Th, and CD4**^+^** IL-17**^+^** Th cells were analyzed by flow cytometry.

### IFN-γ-mediated induction of CXCL9 expression in M1-type Mφs

THP-1 cells in suspension (1×10^5^ cells/ml) were purchased from Noble Biological Co., Ltd. (Shanghai, China). Phorbol ester (PMA) was added to the THP-1 cells in suspension at a final concentration of 50 ng/ml. Then, these cells were seeded into six-well plates (1×10^5^ cells/ml), and adherent mononuclear Mφs were obtained after incubation for 24 h. The cell growth medium was then changed to complete medium without PMA; cells were cultured for 3 days to observe changes in morphology. Pseudopodia were observed, indicating successful Mφs induction.

IFN-γ has been reported to induce Mφs differentiation into M1-type Mφs (15). Here, we stimulated Mφs (1×10^5^ cells/ml) with different concentrations of IFN-γ (0 ng/ml, 1 ng/ml, or 10 ng/ml) for 24 h; we used two experimental methods (i.e., real-time qPCR and ELISA), to measure the CXCL9 expression level at 3 h, 6 h, and 9 h. The primers were synthesized by Jinkairui Biological Engineering Co., Ltd. (Wuhan, China). ELISA kits were purchased from ELK Biotechnology Co., Ltd. (Wuhan, China). Primer sequences are shown in **Supplementary Table S1**.

The Western blot is an important laboratory technique that allows for specific identification and characterization of proteins. Sodium dodecyl sulfate-polyacrylamide gel electrophoresis (SDS-PAGE)-separated proteins are electophoretically transferred to a polyvinylidene fluoride (PVDF) membrane which is then incubated with specific antibodies, then developed to show the protein of interest; β-actin is acted as an internal control. We determined the expression of protein CXCL9, STAT1 and p-STAT1 by Western Blot at different concentrations (IFN-γ 0 ng/ml, 1 ng/ml, or 10 ng/ml) and different times (3 h, 6 h, and 9 h). Reagents were purchased from ASPEN Biotechnology Co., Ltd. (Wuhan, China).

### STAT1-small interfering RNA (siRNA) *in vitro* assay

STAT1-siRNA-CON (negative control group), STAT1-siRNA-1, STAT1-siRNA-2, or STAT1-siRNA-3 were transfected into Mφs (1×10^5^ cells/ml) using Lipofectamine^®^ 2000 (Thermo Fisher Scientific, Waltham, MA, USA); the cells were incubated for 24 h after transfection. The effect of STAT1 interference (STAT1-siRNA-1, STAT1-siRNA-2, and STAT1-siRNA-3) on the expression of STAT1 in Mφs was analyzed by qPCR. The results of STAT1-siRNA interference efficiency verification showed that the three pairs of STAT1-siRNA down-regulated the expression level of STAT1 by 43%, 70%, and 80%, respectively; it met the quality control standards (at least one pair of siRNAs had a silencing efficiency of more than 70% at the mRNA level under standard conditions of use).

IFN-γ (1 ng/ml) was used to stimulate Mφs that had been transfected with STAT1-siRNA-1; after IFN-γ stimulation for 24 h, the level of CXCL9 expression was analyzed by qPCR and ELISA; CXCL9, STAT1 and p-STAT1 protein expression were analyzed by Western blot.

### Measurement of cell migration *via* Transwell assays

CD4**^+^** T cells (1×10^5^ cells/ml) were isolated from the peripheral blood of healthy volunteers using autoMACS columns with the Direct Human CD4**^+^** T Cell Isolation Kit (STEMCELL Technologies). CD4**^+^** T cells were treated with PMA (20 ng/ml) + ionomycin (1 μg/ml) at 37°C for 1 h; Breedellin A (1 μl/ml) was then added and cells were cultured for an additional 3–5 h to obtain Th1 cells.

In the lower chamber of the Transwell plate (Corning, NY, USA), Mφs (1×10^5^ cells/ml) were stimulated with IFN-γ (1 ng/ml) for 24 h; one group was transfected with STAT1-siRNA-1, whereas one group was not transfected. Unstimulated Mφs were used as negative controls. In the upper chamber of the Transwell plate, Th1 cells were seeded. The suspension in the lower chamber was subjected to smear staining to analyze the cell morphology and determine the number of migrating Th1 cells.

### Statistical analysis

Data were stored in a Microsoft Excel-supported database, statistical analyses were performed using SPSS statistics version 20.0 (International Business Machines Corp., NY), and graphics were prepared using GraphPad Prism version 6.0 (GraphPad Software Inc., San Diego, CA). Categorical data of patient characteristics were compared using the Chi-square test. Data with normal distributions were expressed as means ± standard deviations; comparisons among groups were performed by *t*-tests or one-way analysis of variance. Data with non-normal distributions were expressed as medians (interquartile ranges); comparisons between groups were performed by the Mann–Whitney *U*-test. The *Spearman* correlation coefficient was used for correlation analysis. *P*-values <0.05 were considered statistically significant.

## Results

### Demographic data and clinical characteristics

Demographic data and clinical characteristics were collected uniformly from the control and MPP groups (experimental group A and experimental group B). In experimental group A, there were 52 (44.44%) male and 65 (55.56%) female cases, with a male to female ratio of 1:1.25 and a mean age of 5.74 ± 3.03 years.

In experimental group B, there were 8 (34.78%) male and 15 (65.22%) female cases, with a male to female ratio of 1:2.25, while in the control group there were 10 (55.56%) male and 8 (44.44%) female cases, with a male to female ratio of 1.25:1, as shown in **Table 1**. There was no significant difference in terms of sex between the control group and experimental group B (*P>*0.05) and there was a difference in terms of age between two groups (*P*<0.05). Most airway foreign bodies in children occur in children under 3 years old and the peak incidence is in children aged 1-2 years. Therefore, specimens will to be collected to verify the difference level of IFN-γ/CXCL9 between the two groups in the further. The duration of fever was 6.78 ± 2.76 days, the fever peak was 39.36 ± 0.59°C, and the duration of hospitalization was 9.13 ± 2.26 days, as shown in **Table 1**.

### Altered expression of CXCL9 protein in children with MPP

To identify differentially expressed proteins (DEPs) in patients with *M. pneumoniae* infection, we selected six blood samples from the SMPP group, four blood samples from the MPP group, and five blood samples from the control group for protein microarray analysis in a preliminary experiment. DEPs were defined as proteins with *P*<0.05 and fold change >1.2 or <0.83 (absolute log_2_ [fold change] >0.263).

**Figure 1:**
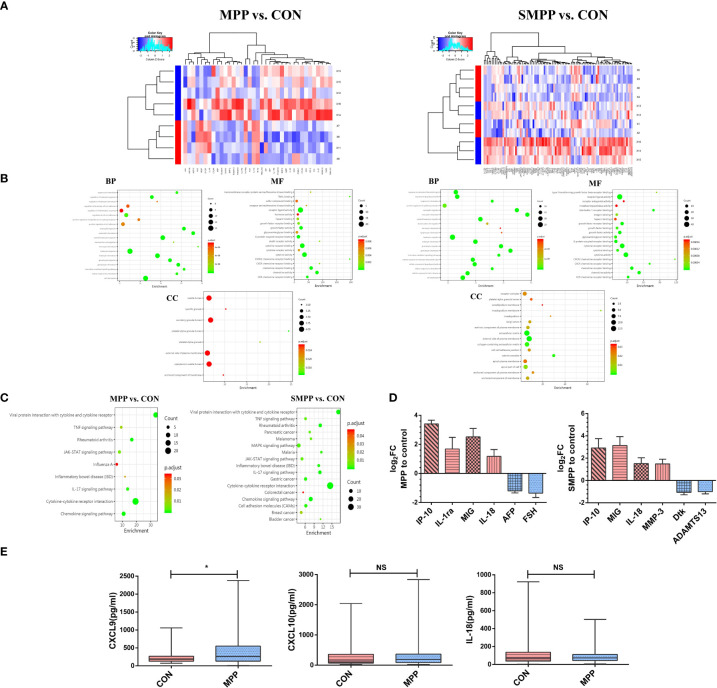
CXCL9 protein expression exhibited the greatest difference between groups (MPP vs. CON; SMPP vs. CON). **(A)** Heatmap of differentially expressed proteins (DEPs) expression abundances (MPP vs. CON: n = 94; 81 downregulated, 13 upregulated; SMPP vs. CON: n = 37; 29 downregulated, 8 upregulated). **(B)** Gene ontology analysis was used to identify the biological functions of DEPs. **(C)** Kyoto Encyclopedia of Genes and Genomes analysis of DEPs revealed enrichment in the cytokine-cytokine receptor interaction pathway. **(D)** Analysis of the first six DEPs by log_2_FC between different groups (MPP vs. CON; SMPP vs. CON; red refers to the up-regulation of expression, blue refers to the down-regulation of expression; MIG: CXCL9; IP-10: CXCL10). **(E)** Comparison of CXCL9, CXCL10, and IL-18 expression between the control and MPP groups during the preliminary experiment; statistical comparison was performed using the Mann–Whitney *U*-test (*P<0.05; NS, not significant).

In terms of DEPs with significant expression, the levels of CXCL9 (MIG), CXCL10 (IP-10), and IL-18 changed between the two groups (**Figures 1A, D**). Gene Ontology (GO) enrichment analysis between the two groups was performed at three levels: cellular component, molecular function, and biological process. DEPs were related to CXCR3 chemokine receptor binding, cytokine response, and cell chemotaxis and migration (**Figure 1B**). Kyoto Encyclopedia of Genes and Genomes (KEGG) enrichment analysis indicated that DEPs might be related to the JAK/STAT signaling pathway (**Figure 1C**). Further clinical pre-experimental research was conducted in the early stage through detecting the same samples by ELISA; only the level of CXCL9 expression significantly differed between the two groups (*P*<0.05) (**Figure 1E**). The level of IFN-γ expression is reportedly closely associated with MPP severity and subsequent recovery; moreover, IFN-γ can induce the production of CXCL9 (24). The above findings suggested that CXCL9 plays an important role in *M. pneumoniae* infection.

### Clinical significance of IFN-γ/CXCL9 in peripheral blood and BALF from children with MPP

**Figure 2:**
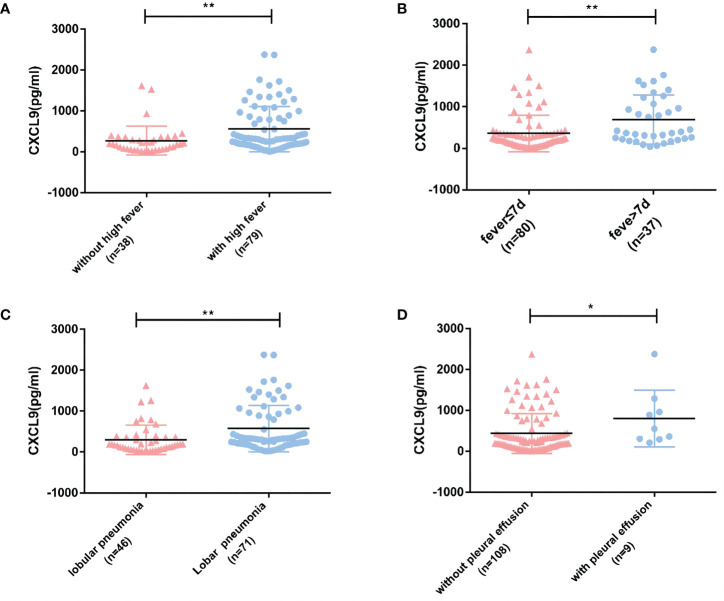
Relationships between CXCL9 expression in peripheral blood and clinical indicators in experimental group A (MPP: n=117). **(A, B)** Relationships between CXCL9 expression and the degree and duration of fever. **(C)** Relationship between CXCL9 expression and the extent of lung lesion on imaging. **(D)** Relationship between CXCL9 expression and pleural effusion. To detect statistically significant differences, the Mann–Whitney *U*-test was carried out (*P<0.05; **P<0.01).

In experimental group A, the duration of fever was 4.52 ± 2.96 days, the fever peak was 39.05(38.70, 39.70) °C. The CXCL9 expression level was upregulated among patients with a higher fever peak, fever duration of greater than 7 days, an imaging manifestation of lobar or segmental, or combined pleural effusion (All *P*<0.05, **Figure 2**).

**Figure 3:**
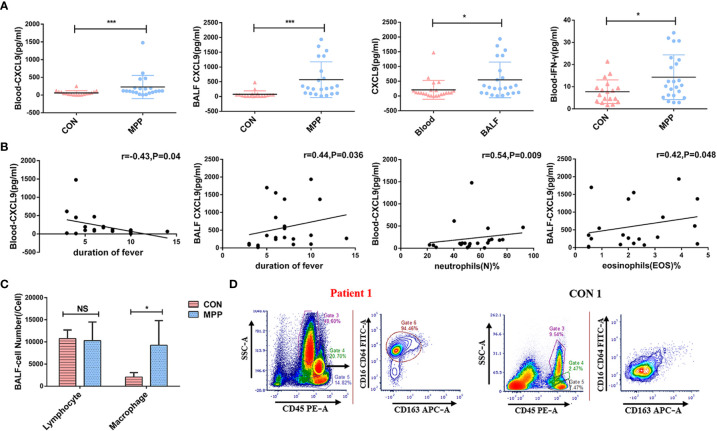
Clinical significance of IFN-γ/CXCL9 in peripheral blood and BALF from children with MPP in experimental group B and control group (MPP: n=23, CON=18). **(A)** Comparison of IFN-γ and CXCL9 expression between groups. **(B)** Correlations between CXCL9 and laboratory indexes. **(C)** Comparison of different types of cells from BALF between groups. **(D)** Flow cytometry result from the BALF between groups of representative cases (patient 1 and CON 1; M1-phenotype Mφs:CD16**^+^** CD64**^+^** CD163**^-^**). Comparisons between groups were performed by the Mann–Whitney *U*-test; correlations between CXCL9 and laboratory indexes were performed using the *Spearman* correlation coefficient; comparison of different types of cells from BALF between groups were performed by *t*-tests. (*P<0.05; ***P<0.001; NS, not significant).

In experimental group B, the peripheral blood levels of IFN-γ and CXCL9, which were higher in patients than in the healthy control group, were positively correlated with each other (*r*=0.502, *P*<0.05). In patients, the CXCL9 expression level was significantly higher in the BALF than in the peripheral blood, and the BALF CXCL9 expression level was higher than that in the healthy control group (All *P*<0.05, **Figure 3A**). The expression level of IFN-γ in BALF between experimental group B and control group was low.

CXCL9 in peripheral blood was negatively correlated with the duration of fever, whereas CXCL9 in BALF was positively correlated with the duration of fever (*r*=-0.43 and *r*=0.44, respectively; both *P*<0.05). CXCL9 in peripheral blood was positively correlated with neutrophils(N)%, whereas CXCL9 in BALF was positively correlated with eosinophils(EOS)% (*r*=0.54 and *r*=0.42, respectively; both *P*<0.05; **Figure 3B**). Other laboratory test data showed no significant correlations, as shown in **Table 1**.

### Cell counts and Mφ phenotypes in BALF from children with MPP

We selected 5 children with MPP in experimental group B and 5 children in the control group to measure cell counts and Mφ phenotypes in BALF by flow cytometry. Results showed that the cell counts of Mφs were significantly higher than those of the control group (*t*=2.844, *P*<0.05; **Figure 3C**). Previous studies have shown that Mφs mainly comprise two distinct functional phenotypes: classically activated Mφs (M1) and alternatively activated Mφs (M2) (25). Our flow cytometry analysis revealed that M1-phenotype Mφs (CD16**^+^** CD64**^+^** CD163**^-^**) were predominant (**Figure 3D**).

### CARDS toxin-mediated differentiation of Th1 cells

**Figure 4:**
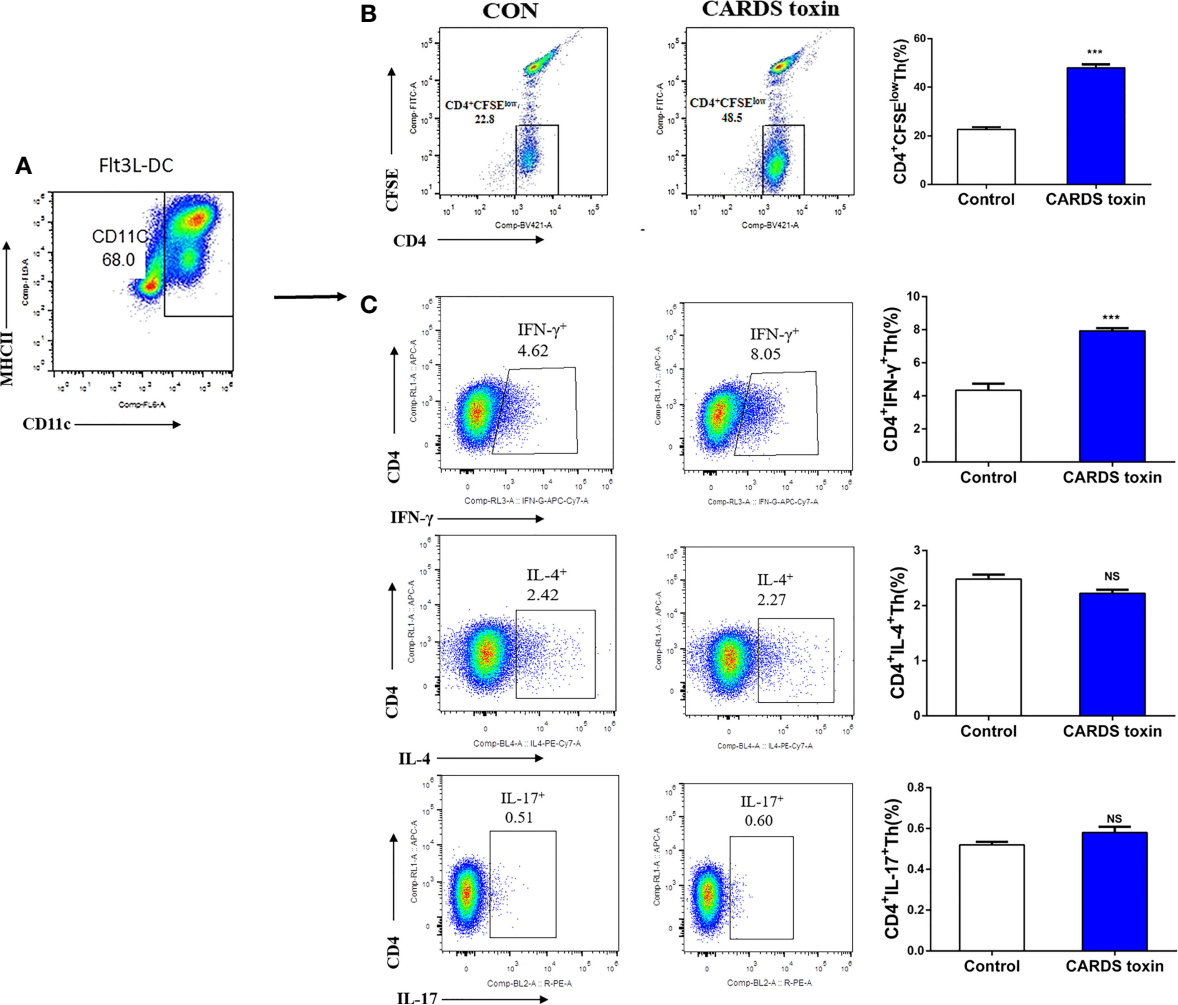
CARDS toxin stimulates F-DCs to regulate Th1 cell differentiation. **(A)** Screening and identification of F-DCs. **(B)** F-DCs were stimulated by CARDS toxin (10 ng/ml) and cell growth medium was used as the negative control; CD4**^+^** T cells with low carboxyfluorescein diacetate succinimidyl ester (CFSE) staining were identified. **(C)** CD4**^+^** IFN-γ**^+^** Th, CD4**^+^** IL-4**^+^** Th and CD4**^+^** IL-17**^+^** Th cells were analyzed by flow cytometry and their proportion among all cells were analyzed between groups (CON vs. CARDS toxin). To detect statistically significant differences, *t*-tests was carried out (***P<0.001; NS, not significant).

Mouse BM-MNCs were isolated, then differentiated into F-DCs using Flt3L; CD11c**^+^** DCs were identified by flow cytometry (**Figure 4A**). CD4**^+^** T cells with low carboxyfluorescein diacetate succinimidyl ester (CFSE) staining were promoted after CARDS toxin stimulation (*P*<0.001, **Figure 4B**). Notably, F-DCs stimulated with CARDS toxin promoted the differentiation of CD4**^+^** IFN-γ**^+^** Th (Th1) cells(*P*<0.001), without affecting the differentiation of Th2 or Th17 cells (**Figure 4C**).

### The IFN-γ-induced high expression of CXCL9 in M1-type Mφs can be blocked by transfection with STAT1-siRNA

**Figure 5:**
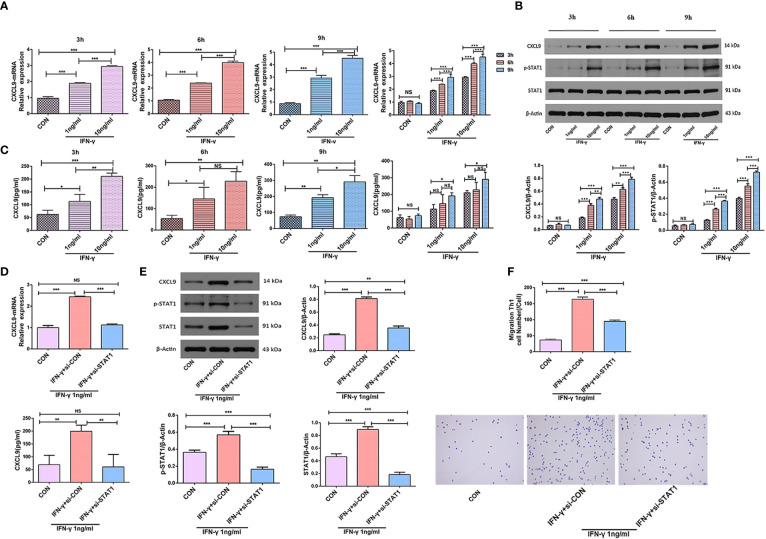
IFN-γ induced CXCL9 expression in a STAT1-dependent manner. **(A)** PMA was added to the THP-1 cells in suspension at a final concentration of 50 ng/ml and mononuclear Mφs were obtained after incubation for 24 h; then, mononuclear Mφs were stimulated with different concentrations of IFN-γ (CON, 1 ng/ml, or 10 ng/ml) and the expression level of CXCL9 was analyzed at different times (3 h, 6 h, 9 h) by qPCR. **(B)** Protein CXCL9, STAT1 and p-STAT1 were analyzed at different times and at different concentrations of IFN-γ by Western Blot. **(C)** The expression level of CXCL9 was analyzed at different times and at different concentrations of IFN-γ by ELISA. **(D)** The expression level of CXCL9 was analyzed by qPCR and ELISA before and after Mφs (IFN-γ 1 ng/ml stimulation) transfection with STAT1-siRNA-1. **(E)** Protein CXCL9, STAT1 and p-STAT1 was analyzed by Western Blot before and after Mφs (IFN-γ 1 ng/ml stimulation) transfection with STAT1-siRNA-1. **(F)** Number of migrating Th1 cells in Transwell experiment and observation of cell morphology by smear staining before and after Mφs (IFN-γ 1 ng/ml stimulation) transfection with STAT1-siRNA-1; Groups: different groups(bottom) + Th1 (top). To detect statistically significant differences, one-way analysis of variance was carried out (*P<0.05; **P<0.01; ***P<0.001; NS, not significant).

IFN-γ promoted the level of CXCL9 expression in a dose-dependent manner at the same time (3 h, 6 h, and 9 h) by qPCR and ELISA (*P*<0.05); qPCR and ELISA also showed that the level of CXCL9 expression significantly increased after stimulation with IFN-γ (1 ng/ml, 10 ng/ml) (*P*<0.05). Upon stimulation with the same concentration of IFN-γ(1 ng/ml), the level of CXCL9 expression significantly decreased after Mφs had been transfected with STAT1-siRNA-1 (*P*<0.05) (**Figures 5A, C, D****)**.

Western Blot showed protein CXCL9 and p-STAT1 were expressed in a dose-dependent and time-dependent manner (*P*<0.05). The expression of protein CXCL9, STAT1 and p-STAT1 increased after stimulation with IFN-γ (1 ng/ml) (*P*<0.05); after Mφs had been transfected with STAT1-siRNA-1, the expression of these proteins significantly decreased (*P*<0.05) (**Figures 5B, E****)**.

### CXCL9 promotes the migration of Th1 cells

The expression of CXCL9 in the lower Transwell chamber increased after 24 h of Mφ (THP-1+PMA induction) stimulation with IFN-γ (1 ng/ml), and the number of migrating Th1 cells increased (*P*<0.001). Whereas, the number of migrating Th1 cells significantly decreased after Mφs had been transfected with STAT1-siRNA-1 (*P*<0.001) (**Figure 5F**).

## Discussion

In recent years, the annual incidence of MPP has been increasing, and some children die because of severe *M. pneumoniae* infection. When it encounters an onslaught of proinflammatory cytokines and cellular elements (e.g., neutrophils, lymphocytes, Mφs, and mast cells) from the host, *M. pneumoniae* strongly adheres to the epithelial cell surface; it uses toxic molecules to damage host cells, thereby inducing ciliostasis and epithelial desquamation to acquire critical nutrients (26). Thus, there is a need to investigate the occurrence and specific regulatory mechanism involved in the immuno-inflammatory response to *M. pneumoniae*; the findings can facilitate early identification and targeted treatment of MPP in children.

In the early stages of this study, protein microarrays from clinical peripheral blood samples showed that CXCL9 was upregulated after *M. pneumoniae* infection. Therefore, the clinical significance of the chemokine CXCL9 in MPP was investigated. CXCL9 is induced by IFN-γ, which is usually secreted by peripheral blood mononuclear cells (24). We found the CXCL9 expression level was upregulated among patients with a higher fever peak, fever duration of greater than 7 days, an imaging manifestation of lobar or segmental, or combined pleural effusion, suggesting that the CXCL9 might have correlation with the disease severity. Our study also showed that the peripheral blood levels of IFN-γ and CXCL9, which were higher in patients than in the healthy control group, were positively correlated with each other. In patients, the CXCL9 expression level was significantly higher in the BALF than in the peripheral blood, and the BALF CXCL9 expression level was higher than that in the healthy control group. Consistent with the findings by Chung et al. (27), that CXCL9 was involved in the pathogenesis of acute respiratory infection involving *M. pneumoniae*. Cytokine release syndrome is regarded as the driver of coronavirus disease 2019 (COVID-2019) inflammation, and multiple studies have demonstrated that CXCL9 is associated with the severity of COVID-2019 (28, 29). Accordingly, CXCL9 may be useful as a clinical biomarker for disease diagnosis and treatment monitoring.

Previous reports (30) concerning experimental models of lower respiratory tract infection with *M. pneumoniae* have indicated that lung disease severity is directly associated with Th1-type cellular immunity; tigecycline treatment significantly reduced the levels of IFN-γ, tumor necrosis factor-α, and IL-1β, CXCL9, and other inflammatory mediators, thereby significantly reducing histological lung inflammation and disease severity. In another study, the ratios of IFN-γ/IL-4 and IFN-γ/IL-13 in BALF are significantly higher in children with MPP than in controls (31), suggesting that children with MPP exhibit a type 1 immune response dominated by Th1-type cellular immunity. Therefore, the type 1 immune response inflammatory factors IFN-γ and CXCL9 are closely associated with lung immuno-inflammatory injury. Furthermore, we found that the level of CXCL9 expression was significantly higher in BALF than in peripheral blood, indicating that lung tissue was the main site of the inflammatory response. IFN-γ stimulates M1-type Mφs to produce CXCL9, which guides Th1 cells along the CXCL9 concentration gradient (from low to high) toward the center of the inflammatory response; this results in a cascade expansion effect that comprises type 1 immune response positive feedback with IFN-γ/CXCL9 as the core circuit, along with a strong immuno-inflammatory response in the lungs.

CARDS toxin, an *M. pneumoniae*-related pathogenic factor, induces cytopathology *in vivo* and *in vitro*, replicates the infection process, and induces histopathological changes similar to *M. pneumoniae* infection; these changes include characteristic fibrillar arrest, cytoplasmic swelling and vacuolization, nuclear fragmentation, extensive inflammation, and histopathological damage (32). As reported (17), the expression level of CARDS toxin increase in the BALF of children with MPP, especially in MPP with mucus plugs and pleural effusion or RMPP. However, the mechanism by which CARDS toxin initiates the subsequent immuno-inflammatory response remains unknown.

Then, through *in vitro* experiments, we explored whether CARDS toxin stimulated F-DCs (dendritic cells incubated with Flt3L) to promote Th-cell differentiation; we also investigated the IFN-γ-induced CXCL9 secretion pathway in macrophages and the role of CXCL9 in promoting Th1 cell migration.

DCs induced by the Flt3L-dependent bone marrow culture system (i.e., F-DCs) have diverse phenotypic characteristics, robust antigen processing, and a strong antigen presentation capacity (33). Therefore, we constructed an F-DC culture identification system to explore the ability of CARDS toxin to stimulate F-DCs to regulate Th cell differentiation; the results showed that F-DCs stimulated by CARDS toxin promoted the differentiation of CD4**^+^** IFN-γ**^+^** Th (Th1) cells without affecting the differentiation of Th2 and Th17 cells. Our findings suggest that CARDS toxin is closely associated with the Th1-type immuno-inflammatory response in the lungs after *M. pneumoniae* infection.

We also found that Mφs increased significantly in BALF after *M. pneumoniae* infection; M1-type Mφs were predominant in BALF from children with MPP. Mφs polarize into different activation states, play different roles, and participate in the progression of different diseases (34). Th1 cell differentiation is reportedly mediated by polarized M1-type Mφs through a mechanism that requires NLRP3 inflammasome activation; IL-1β, IL-6, tumor necrosis factor-α, CXCL9, and CXCL10 are the hallmark cytokines of M1-type Mφs (35, 36). PMA-induced differentiation of THP-1 cells into Mφs is a common *in vitro* model for the analysis of monocyte differentiation (37). Here, we used PMA to induce THP-1 differentiation *in vitro*; we found that IFN-γ promoted the expression of CXCL9 in a dose-dependent manner at the same time. Upon stimulation with the same concentration of IFN-γ, the level of CXCL9 expression increased significantly. These findings showed that the activation of M1-type Mφs was initiated by IFN-γ stimulation; the activated M1-type Mφs then produced CXCL9, Lo et al. (15) reported similar results.

Inflammatory and growth factors often rely on the JAK/STAT signaling pathway to transmit signals that regulate biological effects such as cell growth, proliferation, survival, and inflammatory responses (38). JAK/STAT1 is activated by IFN-γ and plays a central role in Mφ differentiation, maturation, and host defense against pathogen infection (39–41). siRNAs degrade mRNAs with homologous complementary sequences; this loss-of-function mechanism is an important tool for analysis of the roles of genes in biomedical studies (42). To explore the mechanism underlying IFN-γ activation of the CXCL9 signaling pathway, we stimulated Mφs (THP-1+PMA induction) with IFN-γ (1 ng/ml) for 24 h; the level of CXCL9 expression significantly increased. After Mφs had been transfected with STAT1-siRNA-1, the level of CXCL9 expression significantly decreased. These results showed that STAT1-siRNA-1 mediated the reduction of CXCL9 expression in M1-type Mφs by downregulating the level of STAT1 expression. Therefore, the IFN-γ-JAK/STAT1-Mφ-CXCL9 pathway carefully regulates the host immune response in terms of gene and protein expression.

Our study also revealed that CXCL9 was produced in the lower Transwell chamber after Mφs had been stimulated with IFN-γ (1 ng/ml), thus promoting Th1 cell migration. After Mφs had been transfected with STAT1-siRNA-1, the level of CXCL9 expression decreased and the number of migrating Th1 cells significantly decreased. These results confirmed that CXCL9 promoted the migration of Th1 cells, and the migration of Th1 cells was correlated with the concentration of CXCL9.

**Figure 6:**
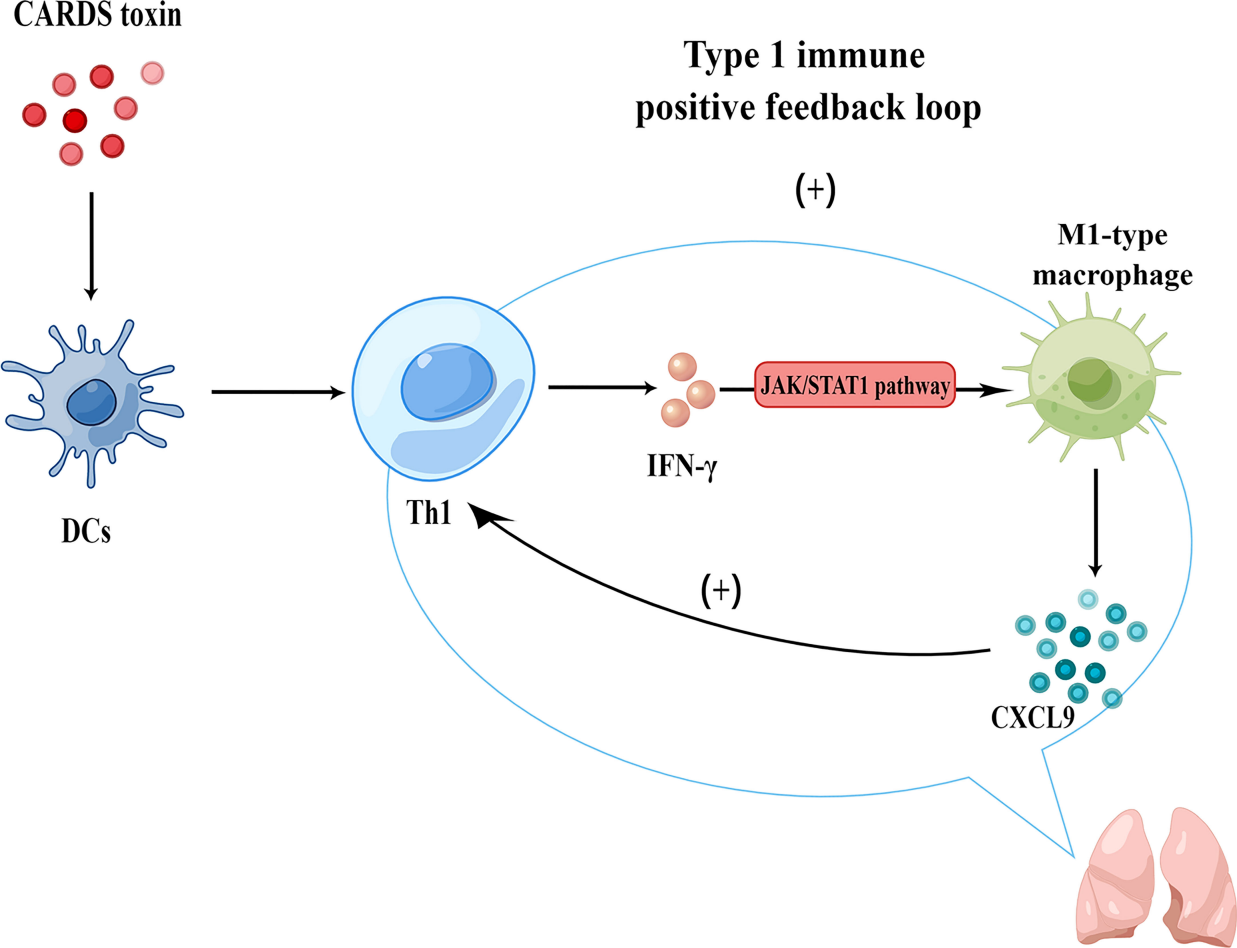
Mechanism of CARDS toxin-induced type 1 immune response positive feedback loop in MPP (generated using Figdraw).

To sum up, CARDS toxin promoted a Th1-type immuno-inflammatory response in the lungs after *M. pneumoniae* infection. Subsequently, IFN-γ activated the JAK/STAT1 signaling pathway and promoted the secretion of CXCL9 by M1-type Mφs; CXCL9 promoted the migration of more Th1 cells to the inflammatory response center, thereby forming a type 1 immune response positive feedback loop that amplified the inflammatory cascade and aggravated immunity-related damage in lung tissue (**Figure 6**).

### Limitations

An important limitation in this study was that we did not characterize the specific mechanism by which CARDS toxin stimulates F-DCs and promotes Th1 cell differentiation. We will continue to focus on this mechanism in future studies.

## Conclusions

Our findings suggest that the level of inflammatory factors IFN-γ/CXCL9 expression can provide new biological markers for early identification, guidance of treatment and prognosis. CARDS toxin induces a type 1 immune response positive feedback loop during *M. pneumoniae* infection; this putative mechanism may be useful in future investigations of immune intervention approaches for *M. pneumoniae* pneumonia.

## Data availability statement

The datasets presented in this study can be found in online repositories. The names of the repository/repositories and accession number(s) can be found in the article/**Supplementary Material**.

## Ethics statement

The studies involving human participants were reviewed and approved by the Ethics Committee of the Children’s Hospital of Soochow University (2019LW014). Written informed consent to participate in this study was provided by the participants’ legal guardian/next of kin. The animal study was reviewed and approved by the Ethics Committee of Soochow University (SUDA20200510A02).

## Author contributions

(I) Conception and design: YY and ZC; (II) Administrative support: YY and ZC; (III) Provision of study materials or patients: TW, HS, ZL, WJ, and GD; (IV) Collection and assembly of data: TW; (V) Data analysis and interpretation: TW, HS, and ZL; (VI) Manuscript writing: All authors; (VII) Final approval of manuscript: YY and ZC. All authors read and approved the final manuscript.

## Funding

This work was supported by Suzhou Livelihood Science and Technology Project (grant NO.SKJY2021104), and Science and Technology Project of Suzhou City (grant NO. KJXW2020025); Social Development Projects of Jiangsu Province (grant NO. BE2019671); the National Natural Science Foundation of China (grant NO. 81870006; 81900006; 82170012); Jiangsu Provincial Medical Youth Talent Project (grant NO. QNRC2016766); Suzhou Medical Youth Talent Project (grant NO. GSWS2019047; GSWS2020053); Suzhou Gusu Health Talents Program (Key Talents) (NO. 2020058); Science and Technology Project of Zhangjiagang City (grant NO. ZKS2108).

## Acknowledgments

We thank Ryan Chastain-Gross, Ph.D., from Liwen Bianji (Edanz) (www.liwenbianji.cn/) for editing the English text of a draft of this manuscript. We gratefully acknowledge the volunteers who participated in our study.

## Conflict of interest

The authors declare that the research was conducted in the absence of any commercial or financial relationships that could be construed as a potential conflict of interest.

## Publisher’s note

All claims expressed in this article are solely those of the authors and do not necessarily represent those of their affiliated organizations, or those of the publisher, the editors and the reviewers. Any product that may be evaluated in this article, or claim that may be made by its manufacturer, is not guaranteed or endorsed by the publisher.

## Supplementary material

Supplementary Table S1 List of primers used in experiments.
