# Supplementary material for: The CARDS toxin of Mycoplasma pneumoniae induces a positive feedback loop of type 1 immune response
Source: Front Immunol. 2022 Dec 1;13:1054788. doi: 10.3389/fimmu.2022.1054788 (PMC9752573; doi:10.3389/fimmu.2022.1054788)
Supplement: Supplementary file 1 [file Table_1.docx]

Supplementary Material

# Supplementary Tables

# Table S1. List of primers used in experiments

| **Gene** | **Sense (5′ → 3′)** | **Antisense (5′ → 3′)** |
| --- | --- | --- |
| β-Actin | GTCCACCGCAAATGCTTCTA | TGCTGTCACCTTCACCGTTC |
| CXCL-9 | CATCATCTTGCTGGTTCTGATT | CTGAATCTGGGTTTAGACATGTT |
| STAT1 | ACTTTCCCTGACATCATTCGC | TCTACAGAGCCCACTATCCGAG |

**
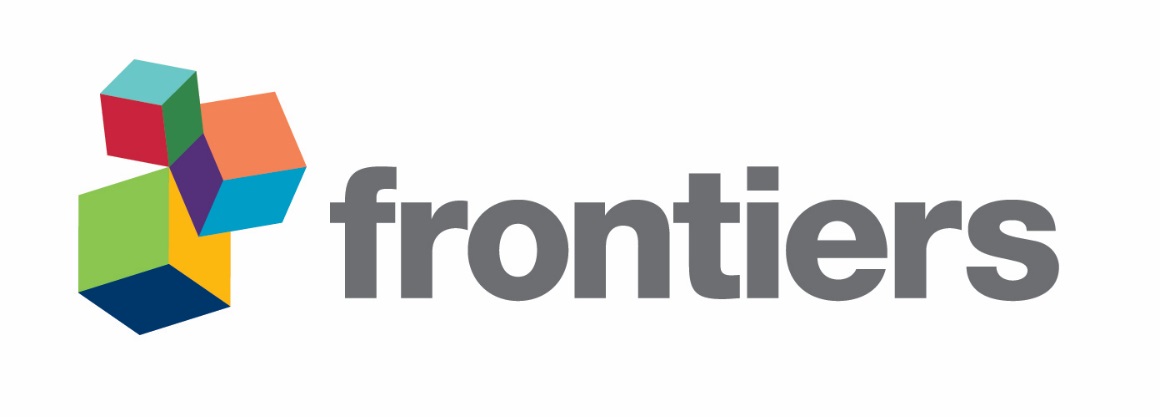
**
