# Supplementary figures and images for: The CARDS toxin of Mycoplasma pneumoniae induces a positive feedback loop of type 1 immune response
Source: Front Immunol. 2022 Dec 1;13:1054788. doi: 10.3389/fimmu.2022.1054788 (PMC9752573; doi:10.3389/fimmu.2022.1054788)

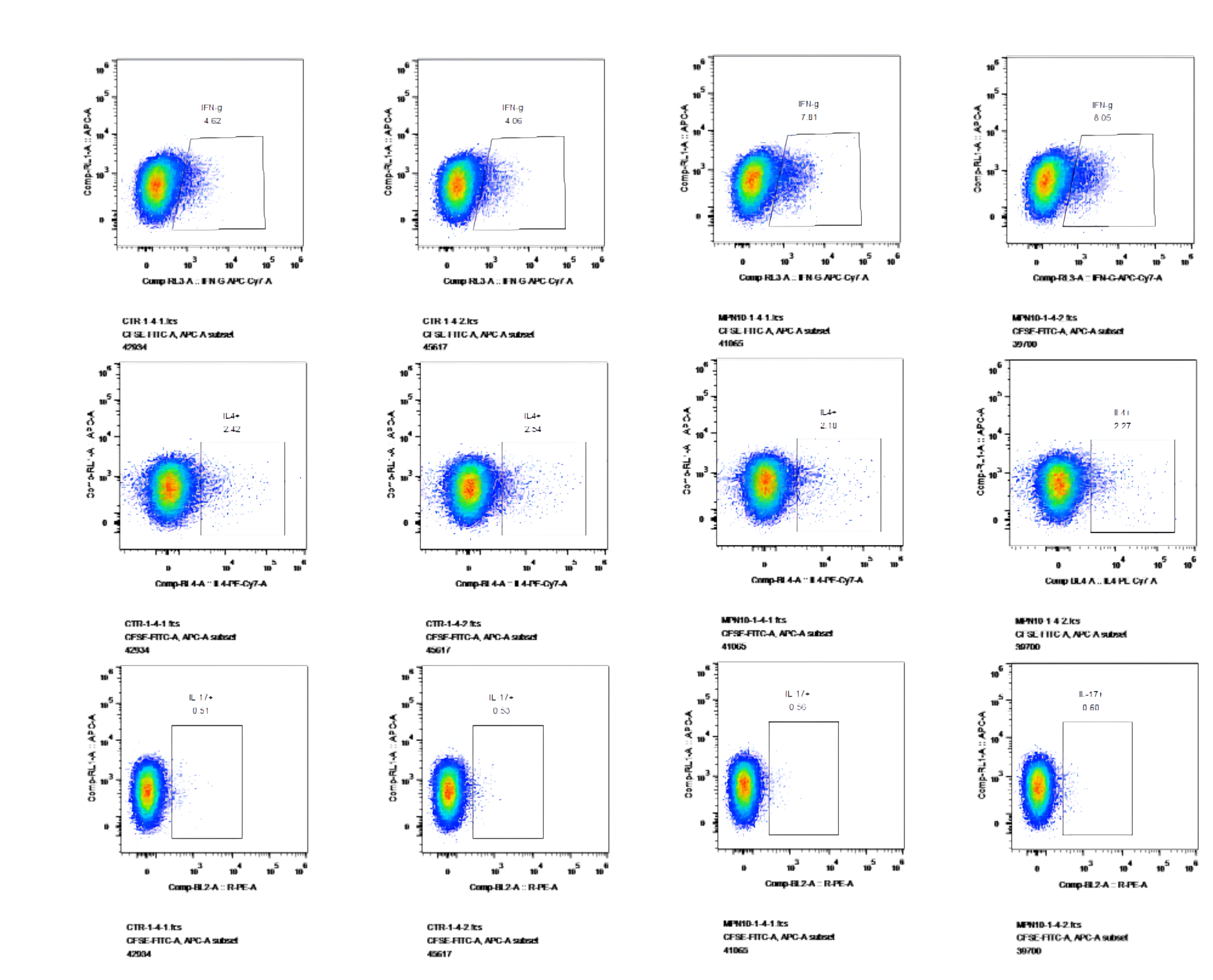

Supplement: Supplementary Table S1 — List of primers used in experiments. [file Image_1.jpeg]
